# A novel small-molecule inhibitor of trefoil factor 3 (TFF3) potentiates MEK1/2 inhibition in lung adenocarcinoma

**DOI:** 10.1038/s41389-019-0173-8

**Published:** 2019-11-04

**Authors:** Mengyi Zhang, Baocheng Wang, Qing-Yun Chong, Vijay Pandey, Zhirong Guo, Ru-Mei Chen, Lingzhi Wang, Yanxin Wang, Lan Ma, Alan P. Kumar, Tao Zhu, Zheng-Sheng Wu, Zhinan Yin, Basappa, Boon-Cher Goh, Peter E. Lobie

**Affiliations:** 1 College of Pharmacy, State Key Laboratory of Medicinal Chemical Biology, Nankai University, Tianjin, China; 2 Department of Pharmacology, Yong Loo Lin School of Medicine, National University of Singapore, Singapore, Singapore; 3 Cancer Science Institute of Singapore, National University of Singapore, Singapore, Singapore; 4 Biomedical Translational Research Institute, Jinan University, Guangzhou, China; 5 Tsinghua Berkeley Shenzhen Institute (TBSI), Tsinghua University, Shenzhen, China; 6 Shenzhen Bay Laboratory, Shenzhen, Guangzhou China; 7 Beijing Chest Hospital, Capital Medical University/Beijing Tuberculosis and Thoracic Tumor Research Institute, Beijing, China; 8 Cancer Program, Medical Science Cluster, Yong Loo Lin School of Medicine, National University of Singapore, Singapore, Singapore; 9 Department of Oncology of the First Affiliated Hospital, Division of Life Sciences and Medicine, University of Science and Technology of China, Hefei, Anhui 230027 China; 10 Department of Pathology, Anhui Medical University, Hefei, Anhui China; 11 Laboratory of Chemical Biology, Department of Studies in Organic Chemistry, University of Mysore, Manasagangotri, Mysore, 570006 Karnataka India; 12 Department of Haematology-Oncology, National University Health System, Singapore, Singapore

**Keywords:** Non-small-cell lung cancer, Target identification

## Abstract

TFF3 has been identified as a novel biomarker to distinguish between lung adenocarcinoma (ADC) and lung squamous-cell carcinoma (SCC). Herein, we determined the oncogenic functions of TFF3 and demonstrated the potential of pharmacological inhibition of TFF3 in lung ADC using a novel small-molecule inhibitor of TFF3 dimerization (AMPC). Forced expression of TFF3 in lung ADC cells enhanced cell proliferation and survival, increased anchorage-independent growth, cancer stem cell behavior, growth in 3D Matrigel, and cell migration and invasion. In contrast, depleted expression of TFF3 suppressed these cellular functions. Mechanistically, TFF3 exerted its oncogenic function through upregulation of ARAF and hence enhanced downstream activation of MEK1/2 and ERK1/2. Pharmacological inhibition of TFF3 by AMPC, resulted in markedly decreased cell survival, proliferation, 3D growth and foci formation, and impaired tumor growth in a xenograft mouse model. Moreover, the combination of various MEK1/2 inhibitors with AMPC exhibited synergistic inhibitory effects on lung ADC cell growth. In conclusion, this study provides the first evidence that TFF3 is a potent promoter of lung ADC progression. Targeting TFF3 with a novel small-molecule inhibitor alone or in combination with conventional MEK1/2 inhibitors are potential strategies to improve the outcome of lung ADC.

## Introduction

Even though significant progress has been made in better staging and greater use of multimodality therapy, lung cancer survival obviously remains very poor with 5-year survival of typically between 6–14% among males and 7–18% among females^1^. Lung adenocarcinoma (ADC) is the most commonly diagnosed lung cancer subtype which accounts for 40% of total lung cancer cases. In addition to surgery, radiation therapy, and chemotherapy, targeted therapies against the well-characterized oncogenic driver mutations in lung ADC, such as EGFR, BRAF, KRAS, and ALK fusion, have been widely administered as ADC treatments^2^. MEK1/2 inhibitors have demonstrated activity in lung ADC patients^3^. Since mutations or amplification of upstream EGFR, BRAF, and KRAS are common in lung ADC and result in constitutive activation of the MAPK pathway, downstream MEK1/2 acts as the unique gatekeeper that can be targeted for inhibition of the MAPK/ERK pathway and tumor progression^4^. Therefore, MEK1/2 inhibitors have been used for treatment of lung ADC both as a monotherapy and in combination with EGFR inhibitors or chemotherapy^5^. Several MEK1/2 inhibitors are approved for use or are currently in the advanced stages of clinical development, with promising prospects in improving survival of lung ADC patients^3^. Nevertheless, the clinical application of targeted therapies have thus far provided limited clinical benefits for lung ADC patients^6,7^.

Accumulating evidence has supported the oncogenic functions of trefoil factor 3 (TFF3) in promoting tumor progression^8–10^. TFF3 expression is increased in a number of cancers, including breast, liver, prostate, gastric, and endometrial as compared with the respective noncancerous tissues^10–14^. The expression of TFF3 has also been reported to be highly associated with metastatic potential of tumors and poor survival outcome of patients in mammary, gastric, and hepatocellular carcinomas^9,10,15^. In normal human lung, immunohistochemistry has revealed significant expression of TFF3 in mucous cells of the acini of submucosal glands and varying amounts in goblet cells^16^, while increased TFF3 expression was observed in bronchioalveolar lavage fluid from patients with chronic obstructive pulmonary disease (COPD)^17^. Levels of TFF3 protein in the serum of lung cancer patients has been reported to be higher than in the serum of healthy individuals^18^. Furthermore, the expression of TFF3 was highly correlated with markers of the adenocarcinomatous lineage in NSCLC^19^. It was observed that >90% of lung ADCs were TFF3-positive, whereas no expression of TFF3 was observed in lung squamous-cell carcinomas (SCC), suggestive of TFF3 as a novel biomarker to distinguish between lung ADC and SCC^19^. Herein, we have elucidated the oncogenic roles of TFF3 in lung ADC, determined the mechanisms involved, and investigated the efficacy of pharmacological inhibition of TFF3 by a novel TFF3 inhibitor (AMPC)^20^, in combination with MEK1/2 inhibitors.

## Materials and methods

### Cell lines and cell culture conditions

NCI-H1299 and NCI-H1975 cell lines from the American Type Culture Collection (Manassas, VA, USA) were kindly provided by Prof. R Soong from the Cancer Science Institute of Singapore. Normal lung epithelial cell line BEAS-2B was provided by Prof. WS Wong from Department of Pharmacology, National University of Singapore. Cell lines used were authenticated and confirmed to be free of mycoplasma and maintained according to ATCC recommendations.

### Transfection and expression analysis

NCI-H1299 and NCI-H1975 cells with forced expression of TFF3 were generated by transfection with empty pIRESneo3 vector or pIRESneo3-TFF3 plasmid and selected in G418 antibiotic as previously described^8^. Stable cells were designated as H1299-Vec/H1299-TFF3 and H1975-Vec/H1975-TFF3 cells respectively. TFF3 in lung ADC cells was transiently depleted using siRNA (s277470 and s14041) from ThermoFisher Scientific (Waltham, MA) as previously described^21^, and the cells were designated as H1299-siSC/H1299-siTFF3 and H1975-siSC/H1975-siTFF3 cells, respectively.

Total RNA isolation, reverse transcription polymerase chain reaction (RT-PCR), and real-time polymerase chain reaction (qPCR) were performed as previously described^8^. Primers sequences used were as previously described^8^. Protein expression was determined by western blot analysis using the primary antibodies listed in Supplementary Methods.

### Cell function assays

The total cell number, cell-cycle analysis, foci formation, colony formation in soft agar, 3D- Matrigel, and transwell migration and invasion assays were performed as previously described^8,22^. Apoptotic cell death was determined using Annexin-V AlexaFluor^®^ 488 Propidium Iodide (PI) Dead Cell Apoptosis Kit (Life Technologies, Gaithersburg, MD) and Caspase-Glo Caspase 3/7 kit (Promega Madison, WI). Spheroid-formation and ALDEFLUOR assays were performed as previously described^10^. The staining of live and dead cells in Matrigel was performed using the LIVE/DEAD^TM^ Cell Imaging Kit (Invitrogen, Carlsbad, CA). Detailed procedures are described in Supplementary Methods.

### Tumor xenograft assays

All animal experiments were performed in accordance with the guidelines for the care and use of laboratory animals at Jinan University (Guangzhou, China). Each 5–6-week-old BALB/c-nu female mouse was injected subcutaneously with 100 μL of cell suspension (1 × 10^7^ cells) in the right flank. Six days after implantation, 24 mice were randomized and divided into three groups, according to treatments administered. In total, 200 μL of vehicle or AMPC was administered by intraperitoneal injection every day for 15 days. The first group of mice (*n* = 8) was treated with vehicle (0 mg/kg; 4% DMSO, 25% PEG400, 50% saline, 21% water); the second and third groups of mice (each *n* = 8) were treated with 20 mg/kg AMPC and 40 mg/kg AMPC, respectively. Twenty-one days after implantation, tumors were excised, photographed, weighed, and fixed as previously described^23^.

### ELISA, histological analysis, and TUNEL assay

TFF3 levels in mice serum were determined by the Human TFF3 DuoSet ELISA kit (R&D systems, Minneapolis, MN). Histological analysis was performed using anti-Ki67 antibody (Abcam, ab15580) and anti-TFF3 antibody (Abcam, ab108599), and stained with hematoxylin (Goodbio Technology, Wuhan, China). Cell apoptosis was detected by terminal deoxynucleotidyl transferase dUTP nick-end labeling (TUNEL) assay using a TUNEL Apoptosis Detection Kit (AlexaFluor 488) (YeSen Biotechnology, Shanghai, China). Detailed procedures are described in Supplementary Methods.

### AMPC, MEK1/2 inhibitors and drug combination assays

AMPC was synthesized in the Department of Studies in Organic Chemistry, University of Mysore^20^. Selumetinib (AZD6244), Pimasertib (AS703026), CI-1040 (PD184352), and Trametinib (GSK1120212) were purchased from MedChemExpress (Monmouth Junction, NJ) and re-constituted in DMSO. Cells were seeded at 2000 cells per well in 96-well plates with 2% FBS media and treated with drug for 48 to 72 h. The viability of cells was determined using AlamarBlue assay (ThermoFisher Scientific, Waltham, MA).

### Statistical analysis

The data were expressed as mean ± S.E.M., and statistical analysis was performed using paired Student’s *t* test. A *p*-value of <0.05 was considered statistically significant. Cell-cycle flow-cytometry results were analyzed using the ModFit LT™ Highlights software. Drug combination index was calculated by the Compusyn software using constant drug concentration ratio. In vitro analysis was performed with three replicates.

## Results

### Forced expression of TFF3 promotes oncogenicity of lung ADC cells

**Fig. 1:**
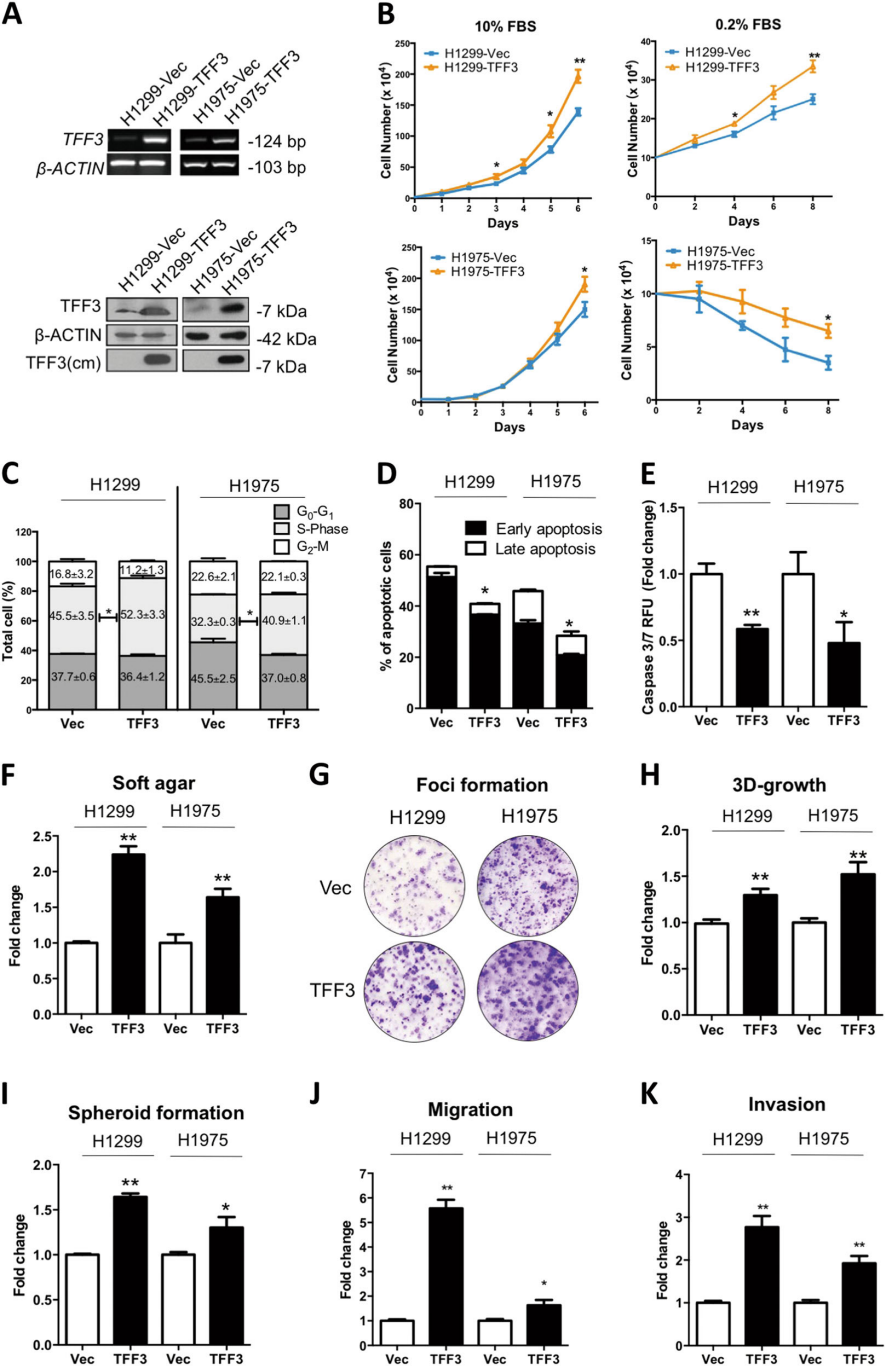
Forced expression of TFF3 promotes oncogenicity of H1299 and H1975 cells. H1299 or H1975 cells were stably transfected with an expression vector containing the TFF3 cDNA (designated H1299-TFF3/H1975–TFF3) or pIRESneo3 vector alone (H1299-Vec/H1975-Vec). **a** Detection of TFF3 expression by RT-PCR and western blot. cm = conditioned media. β-ACTIN was used as input control. **b** The total cell count in RPMI-1640 media supplemented with 10% or 0.2% FBS. Cell numbers were counted at the indicated time points. **c** Cell-cycle progression of cells cultured in 10% FBS medium was determined using propidium iodide (PI) staining followed by FACs analysis. The percentages of cells in each cell-cycle phase are plotted. **d** Annexin-V/PI apoptotic cell death was determined after 24 h serum deprivation. The percentages of early apoptotic (Annexin-V-positive/PI-negative) and late apoptotic (Annexin-V-positive/PI-positive) cells are plotted. **e** Caspase 3/7 activities in the cells were determined after 24 h serum deprivation. **f** Soft agar colony formation. Cells were seeded in 0.35% agarose in full medium on a base layer of 0.5% agarose. Complete culture medium was added and replaced every 3 days. Colonies formed after 14 days were counted and fold change in colony numbers relative to the respective –Vec cells are shown in the histogram. **g** Foci formation. Cells were seeded in six-well plates, and cultured for 2 weeks prior to fixation and crystal violet staining. **h** 3D Matrigel growth. Cells were cultured in 5% FBS medium containing 4% Matrigel. Cell viability was determined by AlamarBlue assay after 9 days. Fold change of cell viability relative to the respective –Vec cells is shown in the histogram. **i** Spheroid-formation assay. Cells were seeded in ultralow attachment plates in spheroid growth media. After 10 days, spheroid growth was measured by AlamarBlue assay, and the fold change of cell viability relative to the respective –Vec cells is shown in the histogram. **j** Cell-migration assay. The number of cells that migrated across the transwell membrane after 12 h were stained with Hoechst 33342 and counted under the fluorescence microscope. Fold change of migrated cells relative to the respective –Vec cells is shown in the histogram. **k** Cell invasion assay. The number of cells that invaded across the 10% Matrigel-coated transwell membrane after 24 h were stained with Hoechst 33342 and counted under the fluorescence microscope. Fold change of invaded cells relative to the respective –Vec cells is shown in the histogram. The data are expressed as mean ± S.E.M. *p < 0.05; **p < 0.01

H1299 and H1975 cells were used as lung non-small cell ADC models due to their moderate mRNA expression of TFF3 based on the results of the expression of TFF3 in 11 human lung ADC cell lines published previously^24^. Thus, the potential oncogenic functions of TFF3 could be determined through both forced or depleted expression of TFF3 in H1299 and H1975 cells. Stable cell lines with forced expression of TFF3, H1299-TFF3, and H1975-TFF3 and respective vector-transfected control cell lines were established. The enhanced expression of TFF3 was verified by RT-PCR and western blot analysis (Fig. 1a).

The effect of TFF3 on cell proliferation and survival was assessed by a total cell number count, cell-cycle analysis, and apoptosis assays, respectively. Forced expression of TFF3 in both H1299 and H1975 cells enhanced cell proliferation. Forced expression of TFF3 in H1299 cells significantly enhanced the total cell number in both full serum (10% FBS) and serum-reduced (0.2% condition) conditions (Fig. 1b). Under serum-reduced conditions, H1975-TFF3 cells maintained a significantly higher total cell number as compared with H1975-Vec cells on day 6, despite the decrease in cell number upon serum deprivation over time. Compared with the H1299/H1975-Vec cells, the percentage of H1299/H1975–TFF3 cells in the S-phase as detected by cell-cycle analysis was significantly increased (Fig. 1c; Supplementary Fig. S1A). Moreover, decreased apoptosis of H1299-TFF3 and H1975–TFF3 cells in serum-free conditions was observed by Annexin-V (Fig. 1d) and Caspase 3/7 assays (Fig. 1e) when compared with the respective vector control cells. Hence, TFF3 promotes lung ADC cell proliferation and survival.

Forced expression of TFF3 has been reported to promote anchorage-independent growth of mammary and hepatocellular carcinoma cell lines^8,10^. Consistently, forced expression of TFF3 in lung ADC cells also enhanced colony formation in soft agar (Fig. 1f; Supplementary Fig. S1B) and foci formation (Fig. 1g). Furthermore, three-dimensional cell culture in Matrigel was used to mimic an in vivo environment^25^. It was observed that forced expression of TFF3 enhanced 3D growth of H1299 and H1975 cells in growth factor-reduced 4% Matrigel (Fig. 1h; Supplementary Fig. S1C). The colonies formed by H1299/H1975–TFF3 cells were significantly larger and acquired an aggressive stellate morphology compared with respective vector control cells.

TFF3 has been reported to be involved in the regulation of cancer stem cell (CSC)-like behavior in cancer^10,21^. To assess the potential function of TFF3 in lung ADC CSC-like behavior, cells were seeded in ultralow attachment plates in serum-free medium supplemented with essential growth factors. Forced expression of TFF3 increased spheroid formation of lung ADC H1299 and H1975 cells (Fig. 1i; Supplementary Fig. S1D).

Transwell assays were performed to determine the migratory and invasive capacity of H1299/H1975-Vec and H1299/H1975–TFF3 cells. It was observed that H1299-TFF3 and H1975–TFF3 cells exhibited a significant increase in migration and invasion compared with H1299-Vec/H1975-Vec cells, respectively (Fig. 1j, k). Similar results were observed in wound-healing assays, with forced expression of TFF3 promoting a more rapid wound closure in H1299-TFF3 and H1975–TFF3 cells compared with their respective control cells (Supplementary Fig. S1E).

### Depleted expression of TFF3 decreases oncogenicity of lung ADC cells

**Fig. 2:**
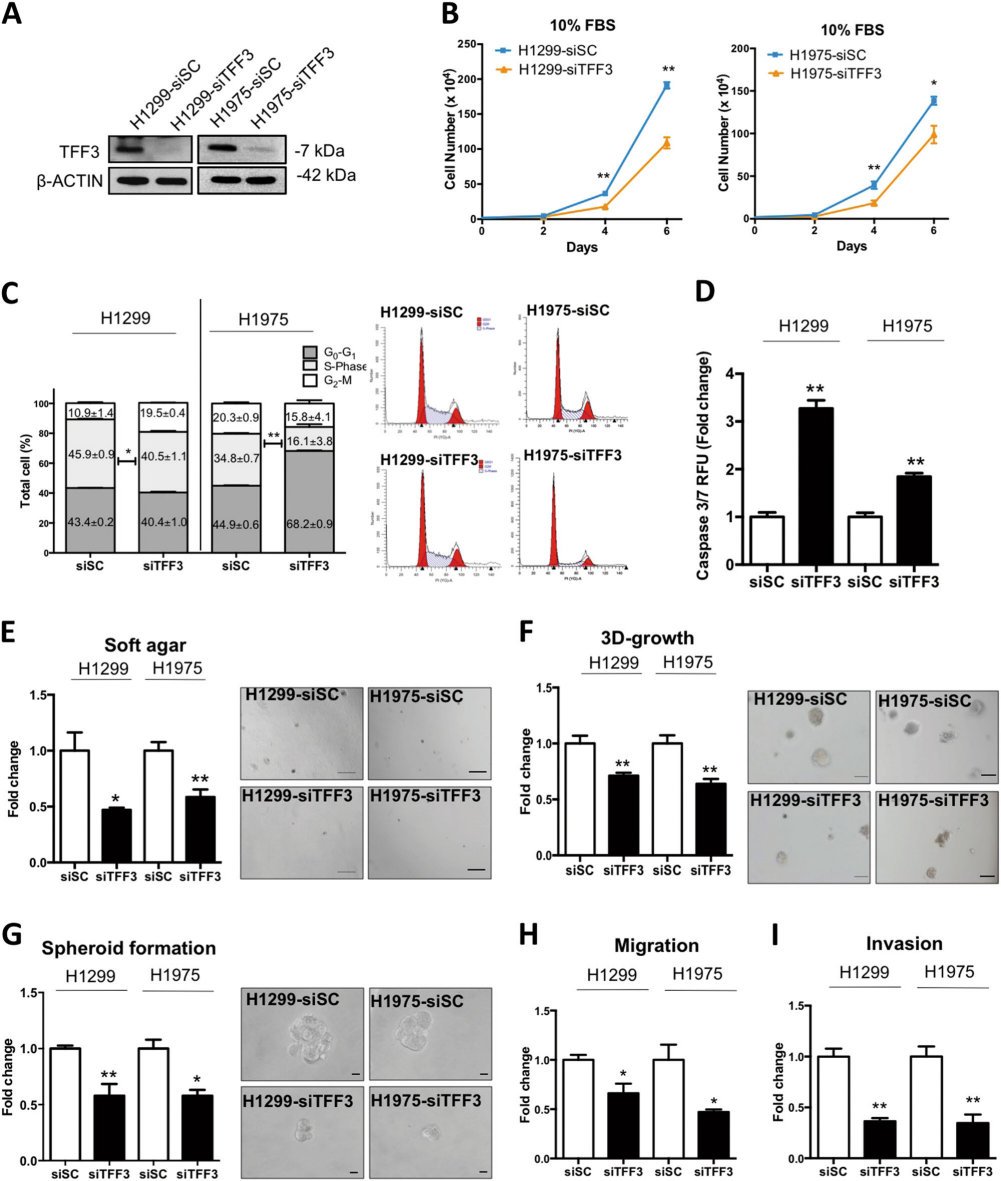
Depleted expression of TFF3 decreases oncogenicity of H1299 and H1975 cells. H1299 or H1975 cells were transiently transfected with TFF3 siRNA (designated H1299-siTFF3/H1975-siTFF3) or scrambled siRNA alone (H1299-siSC/H1975-siSC). **a** Detection of TFF3 expression with western blot analysis. β-ACTIN was used as an input control. **b** The total cell count in RPMI-1640 media supplemented with 10% FBS. Cell numbers were counted at the indicated time points. **c** Cell-cycle progression of cells cultured in 10% FBS medium was determined using PI staining followed by FACs analysis. The percentages of cells in each cell-cycle phase are plotted. **d** Caspase 3/7 activities in the cells were determined after 24 h serum deprivation. **e** Soft agar colony formation. Cells were seeded in 0.35% agarose in full medium on a base layer of 0.5% agarose. Complete culture medium was added and replaced every 3 days. Colonies formed after 14 days were counted, and fold change in colony numbers relative to the respective –Vec cells are shown in the histogram. Representative microscopic images of colonies formed by the respective cells in soft agar are shown. Scale bar: 200 μm. **f** 3D Matrigel growth. Cells were cultured in 5% FBS medium containing 4% Matrigel. Cell viability was determined by AlamarBlue assay after 9 days. Fold change of cell viability relative to the respective –Vec cells is shown in the histogram. Representative microscopic images of colonies formed by the respective cells in 3D Matrigel are shown. Scale bar: 100 μm. **g** Spheroid-formation assay. Cells were seeded in ultralow attachment plates in spheroid growth media. After 10 days, spheroid growth was measured by AlamarBlue, and the fold change of cell viability relative to the respective –Vec cells is shown in the histogram. Representative microscopic images of spheroids formed by the respective cells are shown. Scale bar: 20 μm. **h** Cell-migration. The number of cells that migrated across the transwell membrane after 12 h were stained with Hoechst 33342, and counted under the fluorescence microscope. Fold change of migrated cells relative to the respective –Vec cells is shown in the histogram. **i** Cell invasion assay. The number of cells that invaded across the 10% Matrigel-coated transwell membrane after 24 h were stained with Hoechst 33342, and counted under the fluorescence microscope. Fold change of invaded cells relative to the respective –Vec cells is shown in the histogram. The data are expressed as mean ± S.E.M. *p < 0.05; **p < 0.01

Depleted expression of TFF3 by a well-characterized siRNA^10,21^ was verified by western blot analysis (Fig. 2a). In contrast to forced expression of TFF3 in H1299 and H1975 cells, depleted expression of TFF3 in H1299 and H1975 cells resulted in decreased total cell number compared with control cell lines (Fig. 2b). Cell-cycle analysis showed a decrease in the S-phase fraction of cells in H1299- and H1975-siTFF3 cells compared with H1299 and H1975-siSC (scrambled control) cells (Fig. 2c). In addition, caspase 3/7 activity was increased in H1299- and H1975-siTFF3 cells after 24 h serum starvation compared with scrambled control cells (Fig. 2d).

Depleted expression of TFF3 also significantly decreased anchorage-independent growth as observed by soft agar assay colony formation (Fig. 2e). Furthermore, inhibition of 3D Matrigel growth of H1299- and H1975-siTFF3 cells was observed with TFF3 depletion compared with H1299- and H1975-siSC cells, respectively (Fig. 2f). The spheroid-formation assay also showed a decrease in both the colony size and number, indicating an inhibition of CSC-like behavior with depleted expression of TFF3 (Fig. 2g). In transwell migration and invasion assays, depleted expression of TFF3 decreased both migration and invasion of H1299 and H1975 cells, respectively (Fig. 2h, i). Thus, depleted expression of TFF3 was shown to suppress lung ADC oncogenicity including cell proliferation, survival, anchorage-independent growth, 3D growth, CSC-like behavior, migration, and invasion.

### Pharmacological inhibition of TFF3 decreases oncogenicity of lung ADC cells in vitro

**Fig. 3:**
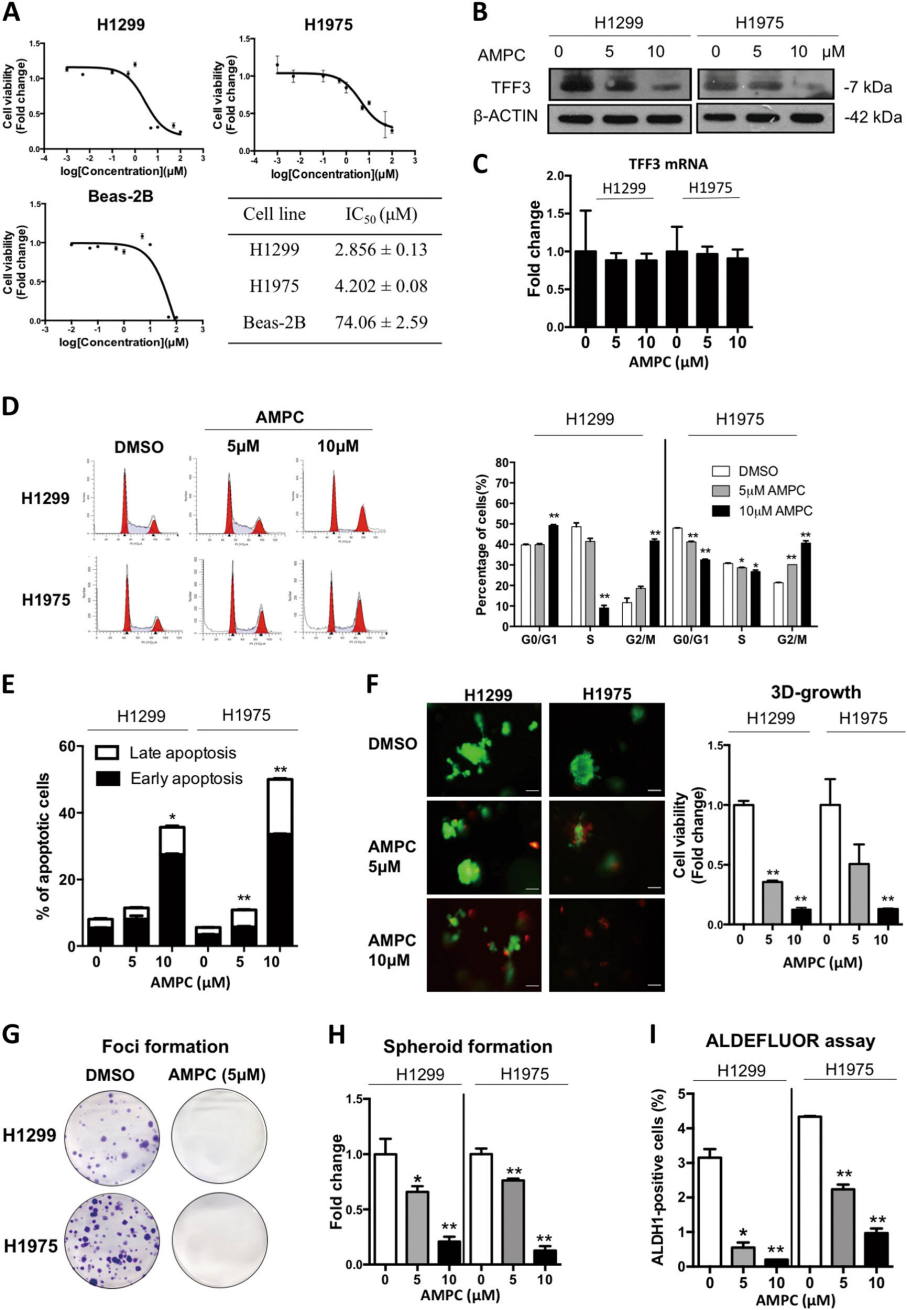
Inhibition of TFF3 by AMPC decreases oncogenicity of H1299 and H1975 cells. **a** Cell viability IC_50_ of AMPC in H1299, H1975, and BEAS-2B cells (Bronchial epithelial cells from normal lung). **b** TFF3 protein levels after AMPC treatment were analyzed by western blot analysis. β-ACTIN was used as an input control. **c** TFF3 mRNA expression levels after AMPC treatment were analyzed by qPCR. β-ACTIN was used as an input control. **d** Cell-cycle analysis after 24 h AMPC treatment. The percentages of cells in each cell-cycle phase are plotted, and statistical significance in the difference in the percentages of AMPC- and vehicle DMSO-treated cells in each phase is shown. **e** Annexin-V/PI apoptotic cell death was determined after 24 h AMPC treatment. The percentages of early apoptotic (Annexin-V-positive/PI-negative) and late apoptotic (Annexin-V-positive/PI-positive) cells are plotted, and statistical significance in the difference in the percentages of apoptotic cells between AMPC- and vehicle DMSO-treated cells is shown. **f** 3D Matrigel growth. Cells were cultured in 5% FBS medium containing 4% Matrigel for 4 days prior to treatment with AMPC for 6 days. Live cells were stained by calcein-AM (green color), and dead cells were stained by PI (red color). Scale bar: 100 μm. The fold change in cell viability after AMPC treatment is shown in the histogram. **g** Foci formation. Cells were seeded in six-well plates, and treated with the indicated concentrations of AMPC for a period of 12 days. The resulting foci formed are fixed and stained with crystal violet. **h** Spheroid-formation assay. Cells were seeded in ultralow attachment plates in spheroid growth media, and treated with the indicated concentrations of AMPC. After 14 days, spheroid growth was measured by AlamarBlue. The fold change in cell viability after AMPC treatment is shown in the histogram. **i** ALDEFLUORTM assay. H1299 and H1975 cells were treated with AMPC for 24 h. The cells were incubated with ALDEFLOUR substrate (BAAA, BoDIPY®-aminoacetaldehyde) to define the ALDH1-positive population while a specific inhibitor of ALDH1 (DEAB) was used to establish baseline fluorescence. The percentages of ALDH1-positive cells after AMPC treatment were determined by flow cytometry. The data are expressed as mean ± S.E.M. *p < 0.05; **p < 0.01

Based on the role of TFF3 in tumor cell survival and metastasis, we rationally designed a small-molecule inhibitor of TFF3, AMPC, specifically to bind to Cys57 of TFF3^20^. It is a first-in-class small-molecule inhibitor of TFF3 that specifically monomerizes dimeric TFF3 to inhibit dimeric TFF3 function^21^. The IC_50_ of AMPC cell viability in H1299 and H1975 was 2.832 (±0.13) μM and 4.277 (±0.08) μM, respectively, whereas the IC_50_ of AMPC in a normal lung epithelial cell line (BEAS-2B) was 78.28 (±2.59) μM. (Fig. 3a). AMPC has previously been demonstrated to reduce cellular TFF3 levels^21^. After treatment of H1299 and H1975 cells with 5 μM or 10 μM AMPC, the levels of TFF3 protein were decreased in both cell lines (Fig. 3b). However, the mRNA level of TFF3 in both cell lines was not affected by AMPC treatment (Fig. 3c).

Consistent with the effect of siRNA mediated depleted expression of TFF3, inhibition of TFF3 by AMPC decreased cell proliferation, and induced apoptosis in lung ADC cells. AMPC treatment for 24 h at concentrations of 10 μM significantly decreased cell cycle S-phase and increased cell cycle G2-phase over 20% in H1299 and H1975 cells (Fig. 3d). Inhibition of TFF3 by AMPC in H1299 and H1975 cells increased the proportion of the cell populations in both early apoptosis and late apoptosis in a dose-dependent manner (Fig. 3e; Supplementary Fig. S2A). In addition, treatment of AMPC significantly inhibited H1299 and H1975 cell anchorage-independent growth in soft agar colony-formation and foci formation assays, wherein 5 μM AMPC completely inhibited foci formation in H1299 and H1975 cells (Fig. 3g; Supplementary Fig. S2B).

We next determined the effect of AMPC on the 3D Matrigel growth of H1299 and H1975 cells. Calcein green and propidium iodide (PI) were used to stain live and dead cells, respectively, in the colonies formed by H1299 and H1975 cells in 3D Matrigel to visualize cell death consequent to AMPC treatment. Colonies were preformed and the treatment with AMPC started on the 4th day, and continued every 2nd day until the 10th day. Live colonies (calcein-AM, green color) were significantly decreased whereas the proportion of dead cells (propidium iodide, red color) was increased (Fig. 3f). In addition, we observed that AMPC treatment of both cell lines dramatically decreased total cell viability in 3D culture consistent with the visualization of live/dead colonies from the calcein green/PI staining (Fig. 3f).

We further examined whether the pharmacological inhibition of TFF3 would affect the CSC-like population in lung ADC cells. Inhibition of TFF3 by AMPC significantly decreased the number and size of spheroids generated by both H1299 and H1975 cells (Fig. 3h; Supplementary Fig. S2C). To further determine the effect of AMPC on CSC-like behavior, aldehyde dehydrogenase-1 (ALDH1), an established cancer stem cell marker, was assessed in H1299 and H1975 cells by the ALDEFLUOR^TM^ assay^24^. We observed a 93.3% (from 3.0% to 0.2%) and 74.7% (from 4.3% to 1.1%) decrease of the ALDH1-positive population in H1299 and H1975, respectively, after treatment with 10 μM AMPC (Fig. 3i; Supplementary Fig. S3A). We have also observed that several cancer stem cell related genes were regulated by forced expression of TFF3 in H1299 cells or by AMPC treatment of H1299 cells. The mRNA expression of ALDH1, CD133, ALCAM, and LGR5 were significantly and consistently increased by forced expression of TFF3 in H1299 cells and decreased by AMPC treatment of H1299 cells compared with vehicle-treated control cells (Fig. S3B). In addition, treatment of H1299 cells with AMPC decreased the protein expression of ALDH1, CD133, ALCAM, LGR5, and c-Myc (Supplementary Fig. S3C). In summary, AMPC is a potent and effective inhibitor of cell proliferation, survival, 3D growth, and CSC-like behavior in lung ADC cells with TFF3 expression.

### AMPC suppresses lung ADC tumor growth in a mouse xenograft model

**Fig. 4:**
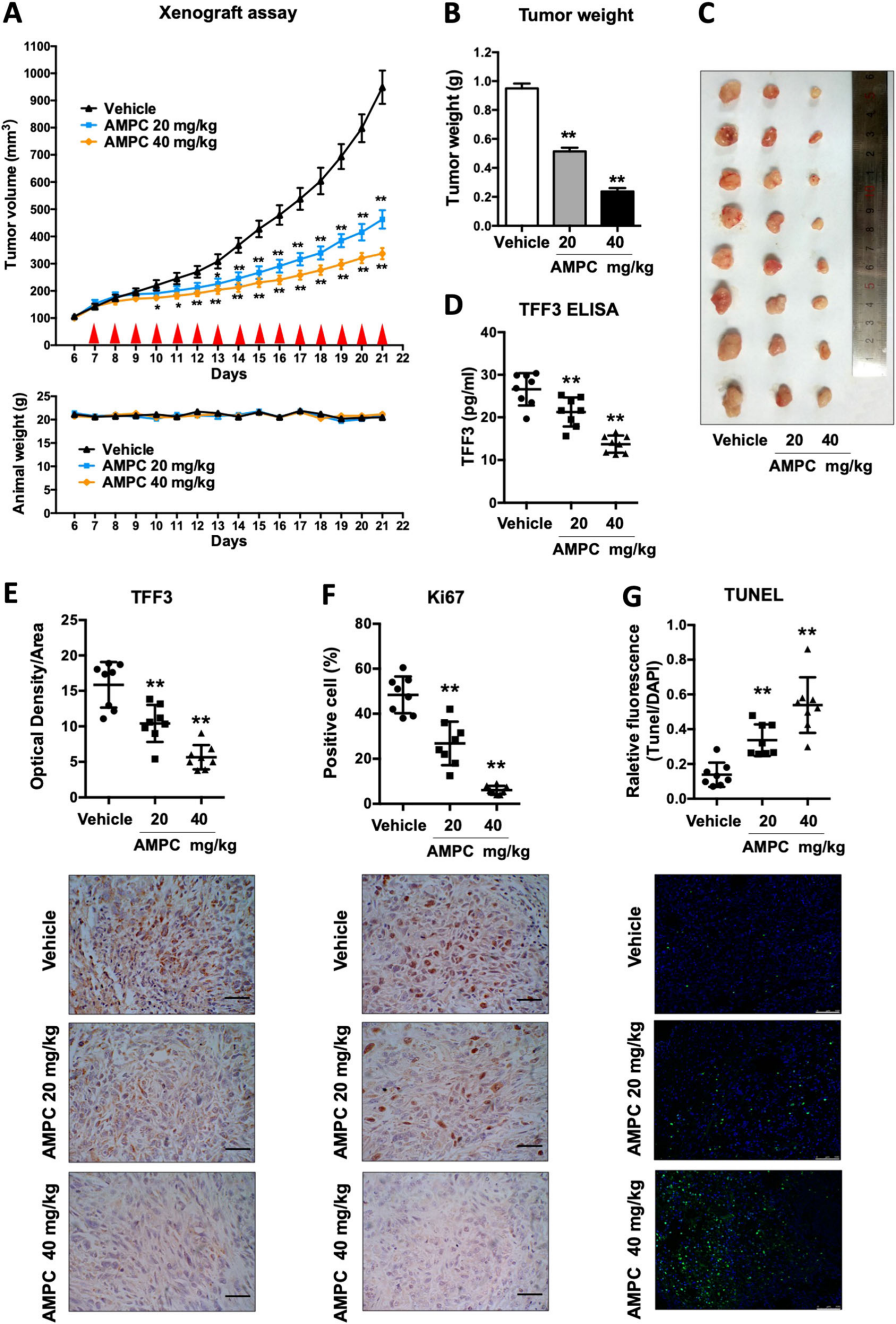
Inhibition of TFF3 by AMPC inhibits tumor growth of H1975 xenografts. **a** Female nude mice were orthotopically injected with H1975 cells, and palpable lung tumors were observed after 6 days. These mice were randomized into three groups (*n* = 8 for each group) that were treated with the vehicle, 20 mg/kg AMPC or 40 mg/kg AMPC starting on day 7 following implantation. Red triangles represent timing of treatment. Mean tumor volumes were recorded and graphically represented. The weights of the mice were measured daily for the whole duration of the experiment. **b** Tumor weight. Tumors were excised and weighed 21 days after implantation. Mean tumor weights in each group are shown in the histogram. **c** Resected tumors from the mice in each group. **d** Serum TFF3 concentrations from vehicle or AMPC-treated mice were determined by ELISA and represented in a scatter plot. **e** Histological staining for TFF3 in tumors from the three groups of mice. Staining intensities of the tumor sections are shown in the scatter plot, and representative image of TFF3 IHC staining in a tumor from each group is shown. Scale bar: 50 μm. **f** Histological staining for Ki67 in tumors from the three groups of mice. The percentages of Ki67-positive cells in the tumor sections are shown in the scatter plot, and representative image of Ki67 staining in a tumor from each group is shown. Scale bar: 50 μm. **g** TUNEL assay. TUNEL-positive nuclei as indicated by fluorescence represent apoptotic cells. The percentages of apoptotic cells in the tumor sections are shown in the scatter plot, and representative image of TUNEL-positive cells in a tumor from each group is shown. Scale bar: 50 μm. The data are expressed as mean ± S.E.M. *p < 0.05; **p < 0.01

To determine if AMPC would inhibit in vivo tumor growth, we established a xenograft model via subcutaneous injection of H1975 cells in immunocompromised mice. Once tumor volume reached ~150 mm^3^ on average the mice bearing the H1975 xenograft tumors were randomly grouped into three groups and injected intraperitoneally with either control vehicle, AMPC at 20 mg/kg or 40 mg/kg once daily for 15 days. A significant reduction of tumor volume in drug-treated mice was observed as compared with the vehicle-treated counterparts from day 7 for the 20 mg/kg treated group and day 4 for the 40 mg/kg treated group (Fig. 4a). During the 15-day treatment period, animal weight remained stable, indicative that the drug treatment was well tolerated (Fig. 4a, below). At the end of day 21, the mice were sacrificed and tumors were harvested for further analysis. The average tumor weight of the 40 mg/kg AMPC-treated group was observed to be approximately four fold less than that of the vehicle-treated group, and 20 mg/kg group was approximately 1.9 fold less than the vehicle-treated group (Fig. 4b, c).

Consistent with the effects of AMPC in vitro, we also observed from immunohistochemical analysis that TFF3 expression was decreased in a dose-dependent manner in tumors from AMPC-treated mice (Fig. 4e). TFF3 is a secreted protein, and the levels of TFF3 in serum were also significantly decreased in the AMPC treatment groups in a dose-dependent manner compared with the vehicle control group, indicative that serum TFF3 could be further developed as a response marker for the efficacy of AMPC treatment (Fig. 4d). Histological analysis also demonstrated that AMPC treatment resulted in a significant decrease of Ki67 labeling and increased apoptosis as observed by TUNEL assay, consistent with the in vitro observations (Fig. 4e, f).

### TFF3 increases ARAF expression with resultant activation of the MAPK/ERK pathway

**Fig. 5:**
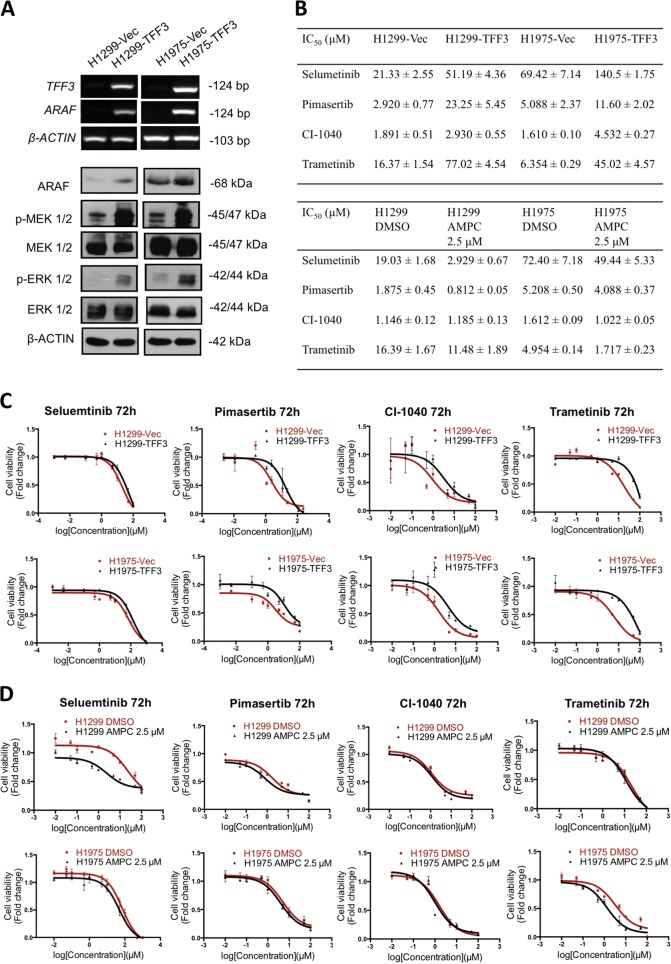
TFF3 increases ARAF expression with resultant activation of the MAPK/ERK pathway **a** Detection of ARAF mRNA levels by RT-PCR, and the expression and activation levels of the proteins in the MAPK/ERK pathway by western blot analysis, β-ACTIN was used as an input control. **b** IC_50_ values of MEK1/2 inhibitors in H1299 and H1975 cells, either with forced expression of TFF3 or AMPC inhibition of TFF3, cultured in media supplemented with 2% FBS at 72 h. **c** Dose response curves of MEK1/2 inhibitors in H1299-VEC, H1299-TFF3, H1975-VEC, and H1975–TFF3 cells. **d** Dose response curves of MEK1/2 inhibitors, in combination with either 2.5 µM AMPC or vehicle DMSO, in H1299 and H1975 cells. The data are expressed as mean ± S.E.M.

To delineate the critical pathways regulated by TFF3 in lung ADC cells, we used qPCR to examine the effect of TFF3 on the expression of genes involved in cell-cycle progression, apoptosis, senescence, adhesion, invasion, and related signal transduction molecules (Supplementary Table S1). Among these genes, we observed that the proto-oncogene *ARAF* was the gene most highly regulated by forced expression of TFF3 in lung ADC cells. Consistent increases in the mRNA level of ARAF were also observed in H1975–TFF3 and H1299-TFF3 cells compared with the respective control cell lines by RT-PCR (Fig. 5a), as were increases in ARAF protein by western blot analysis (Fig. 5a). ARAF protein was also decreased by treatment of both cell lines with AMPC (Supplementary Fig. S3D). ARAF, a proto-oncogene belonging to the RAF subfamily of the Ser/Thr protein kinase family, has been reported to be involved in cell proliferation and survival through the Ras/MEK/MAP kinase signal transduction pathway^26,27^. Consequently, forced expression of TFF3 in H1299 and H1975 cells resulted in enhanced activation of both MEK1/2 and ERK1/2 compared with the respective control cells (Fig. 5a).

Current strategies for targeting the RAR/MEK/MAPK kinase pathway focus on inhibition of downstream effector molecules including MEK1/2 and ERK1/2. MEK1 and MEK2 are considered as gatekeepers of the MAPK/ERK pathway, as the only known activators of ERK1/2^3^. Preclinical investigations also suggest that inhibition of MEK1/2 could be an effective strategy for the treatment of tumors driven by upstream BRAF or KRAS mutations^5,28^. We therefore examined the effect of forced expression of TFF3 in H1299 and H1975 cells on the efficacy of four commercially available MEK1/2 inhibitors, namely Selumetinib, Pimasertib, CI-1040, and Trametinib. The IC_50_ of the four MEK1/2 inhibitors were consistently higher in H1299-TFF3 and H1975–TFF3 cells compared with the control cell lines (Fig. 5b, c). In contrast, significantly decreased IC_50_ values of the MEK1/2 inhibitors in both H1299 and H1975 cells were achieved when the cells were treated with 2.5 µM AMPC simultaneously (Fig. 5b, d) (except for CI-1040 in H1299 cells). The IC_50_ reduction of Selumetinib and Pimasertib in H1299 cells were 6.5-fold and 2.3-fold, respectively, suggesting that inhibition of TFF3 by AMPC in lung ADC cells augments the sensitivity of lung ADC cells to MEK1/2 inhibitors.

### Synergistic combination effects between AMPC and MEK1/2 inhibitors in lung ADC cells

**Fig. 6:**
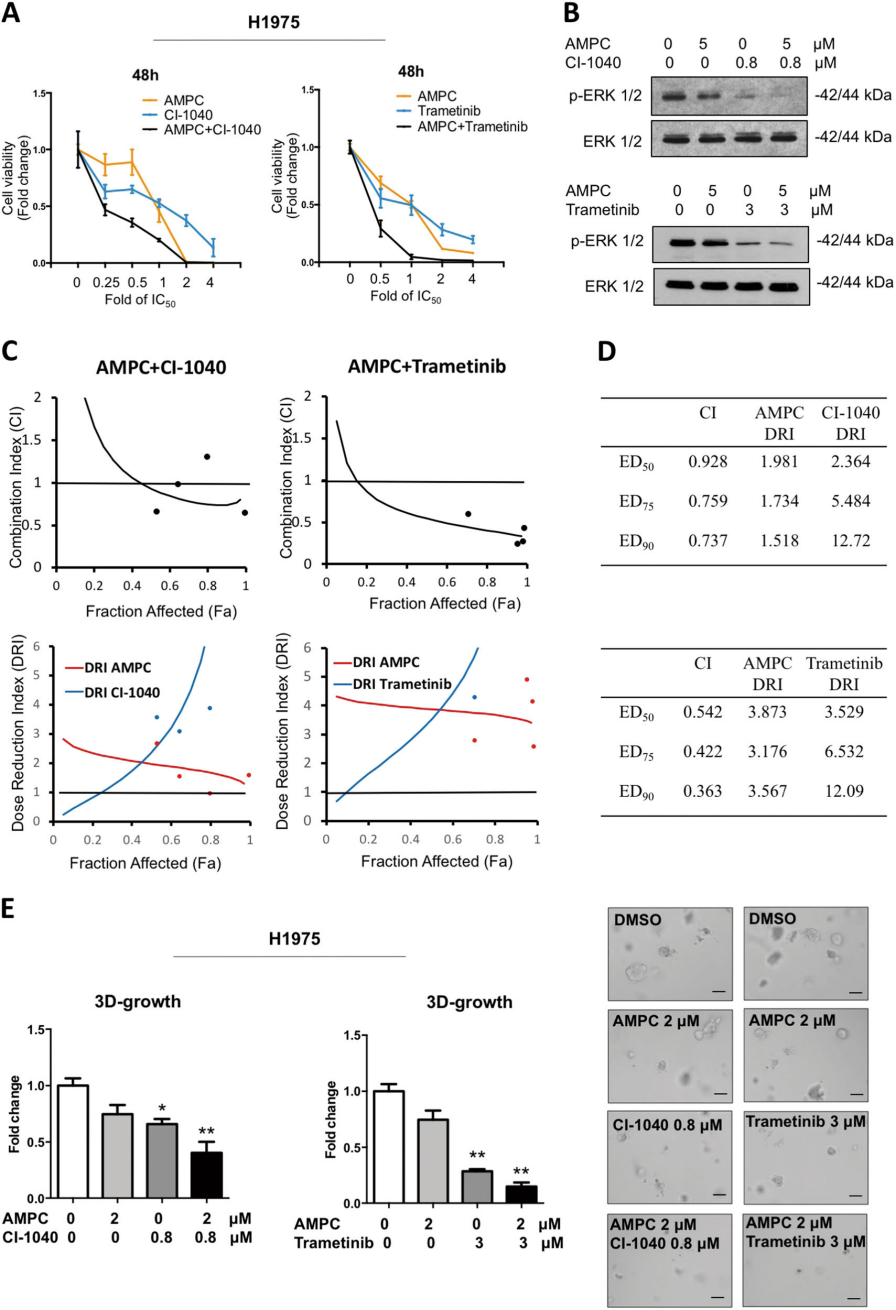
Synergistic combination effects between AMPC and MEK1/2 inhibitors in H1975 cells. **a** Drug combination of AMPC and MEK1/2 inhibitors. H1975 cells were seeded in 96-well plate and treated with AMPC, CI-1040/trametinib or the combination at different concentrations for 48 h. Fold change in cell viability is plotted. **b** Western blot analysis of p-ERK and ERK protein levels in cell lysates of H1975 treated with AMPC, CI-1040/Trametinib alone or the combination for 24 h. The levels of the total ERK was used as an input control. **c** Combination index (CI) and dose-reduction index (DRI) plots of AMPC and CI-1040/trametinib combination treatment in H1975 cells. **d** CI and DRI values at ED_50_, ED_75_, and ED_90_ of AMPC and CI-1040/trametinib combination treatment in H1975 cells. **e** 3D Matrigel growth. H1975 cells were seeded in 5% FBS media containing 4% Matrigel and allowed to form colonies for 4 days prior to treatment with AMPC, CI-1040/trametinib or the combination for 7 days. Fold change in cell viability is plotted. Representative microscopic images of colonies formed by H1975 cells treated with AMPC, CI-1040/trametinib or the combination in 3D matrigel. Scale bar: 100 μm

Drug combinations generally produce improved therapeutic outcomes compared with single-agent treatment^29^. Selumetinib and Trametinib are FDA approved, whereas several other MEK1/2 inhibitors are at different stages of clinical development^3^. Among these agents, Trametinib has the greatest affinity for the MEK1/2 allosteric site, and has been approved for advanced NSCLC patients with BRAF^V600E^ mutation in combination with Dabrafenib^3,30^. We examined the effect of AMPC in combination with the four MEK1/2 inhibitors in both H1299 and H1975 cells at 48 h and 72 h (Supplementary Table S2). Overall, the combination effect of AMPC with Selumetinib or Pimasertib was additive, but the combination of AMPC with CI-1040 or Trametinib in H1299 and H1975 exhibited synergistic effects in terms of reduction of cell viability based on the Chou–Talalay analysis (Fig. 6a; Supplementary Table S2).

The combination of AMPC and CI-1040 or Trametinib in H1975 cells resulted in a greater decrease in ERK1/2 activity, consistent with the enhanced reduction in cell viability observed (Fig. 6b). Analysis by Compusyn software showed drug combination index (CI) and dose-reduction index (DRI) across a range of different dose levels (Fig. 6c, d). The average CI value from ED_50_ to ED_90_ was 0.801 for the combination of AMPC and CI-1040, and the average CI value was 0.447 when AMPC was combined with Trametinib in H1975 cells. The DRI value also suggested a greater than threefold dose reduction when AMPC was combined with Trametinib. Furthermore, a greater drug-induced inhibition of H1975 cell growth in Matrigel was observed after treatment with the combination of AMPC with CI-1040 or Trametinib (Fig. 6e).

## Discussion

Herein, we provided functional and mechanistic evidence that TFF3 promotes tumor progression in lung ADC, following the previous observation of highly specific TFF3 expression in lung ADC^19^. This is corroborated by the previously reported oncogenic functions of TFF3 in mammary, prostate, hepatocellular, endometrial, and cervical carcinomas^8,10,14,31,32^. Furthermore, TFF3 has been implicated in reduced sensitivity and acquired resistance to chemo-, hormonal, and targeted therapies in different cancers; and in particular, mammary carcinoma with acquired trastuzumab resistance exhibited a shift in oncogene addiction from HER2 to TFF3^8,10,21^. As TFF3 exhibits increased expression in various cancers to promote cancer progression, therapeutic resistance, and relapse, and is associated with poor patient survival, it is a potential actionable target in cancer^9,11–13,33,34^. Hence, we have designed a novel small-molecule inhibitor of TFF3, AMPC (Supplementary Fig. S4), which specifically targets the Cys57 residue of TFF3 and monomerizes dimeric TFF3^20^. It was previously shown that TFF3 homodimers are required for its anti-apoptosis function^35^. Hence the AMPC-mediated monomerization of TFF3 inhibits the crucial anti-apoptotic functions of TFF3, which is consistent with our observations in lung ADC herein. It has been observed that monomeric TFF3 exhibits a markedly shorter half-life compared with dimeric TFF3, accounting for the observation of decreased TFF3 protein levels upon AMPC treatment in lung ADC cells. Concordantly, the in vivo study showed a decrease in TFF3 levels in mice serum upon AMPC treatment, suggestive that serum TFF3 levels may be a promising biomarker for the clinical response to AMPC.

Recent studies have shown that TFF3 promotes CSC-like behavior in mammary and hepatocellular carcinoma cells^10,21^, consistent with the current study in lung ADC. Accordingly, the inhibition of TFF3 by AMPC in lung ADC significantly decreased the CSC-like population. CSCs are responsible for cancer development, tumor cell self-renewal, generation of unlimited progeny, metastasis, and resistance to therapy^36^. Importantly, the existence of CSCs is believed to be an important mechanism of drug resistance^37^, and TFF3-induced cancer stem cell-like behavior has been shown to contribute to chemoresistance (doxorubicin) in hepatocellular carcinoma and trastuzumab resistance in HER2+ /ER+ mammary carcinoma^10,21^. Hence, the pharmacological inhibition of TFF3 by AMPC through inhibition of CSC-like behavior may be further explored as one potential mechanism to improve drug sensitivity in human lung ADC.

TFF3 is suggested to function as a promiscuous ligand, being involved in several signaling pathways that contribute to oncogenesis^21,38^. To date, CXCR4/7 is the only receptor reported to bind TFF3 directly albeit in monomeric form and at low affinity, yet TFF3-mediated cell proliferation and ERK1/2 activation was shown to be independent of this receptor^38^. TFF3 was also reported to indirectly activate HER family (HER1–4), cMET, and insulin-like growth factor 1 receptor (IGF1R), which are receptors regulating downstream pathways, including MAPK/ERK signals^21^. The forced expression of TFF3 was observed to promote activation of AKT in hepatocellular carcinoma cells, while inhibition of AKT abrogated TFF3-mediated increase in oncogenicity and chemoresistance of hepatocellular carcinoma cells^10^. Moreover, TFF3 has been demonstrated to promote the activation of c-Src, a non-receptor tyrosine kinase that activates ARAF^9,39^. ARAF, together with CRAF, has previously been shown to induce ERK1/2 phosphorylation and Gl/S cell-cycle progression, consistent with our observation that TFF3 promotes cell-cycle progression in lung ADC^40^. It has been reported that TFF3 acts through EGFR to activate downstream pathways including p44/42MAPK, while the inhibition of p44/42MAPK specifically abrogated migratory properties of oral squamous-cell carcinoma cells stimulated by exogenous TFF3^41,42^.

RAS/MEK/MAPK functions downstream of EGFR and HER2 signaling and mediates various oncogenic behaviors, including cell proliferation, survival, migration, and drug resistance^43^. The increased expression of EGFR or mutations in the intracellular domain of the EGFR have been reported in 43–89% of NSCLC;^44,45^ BRAF mutations were observed in 2–4% of lung ADCs;^46^ and KRAS mutations were detected in 20–30% of NSCLC^47^. Hence, MEK1/2 inhibitors have been considered as a potential therapy in lung cancer with acquired mutations in *EGFR*, *KRAS*, or *BRAF*^29,48,49^, and have been actively studied in clinical trials^3^. CI-1040 is an orally active, highly specific, small-molecule inhibitor of MEK1/2 and was generally well tolerated in the phase II study with advanced NSCLC^50,51^. Trametinib is a FDA-approved drug for the treatment of BRAF^V600E^ or ^V600K^ mutation-positive unresectable or metastatic melanoma, advanced anaplastic thyroid cancer, or metastatic NSCLC together with dabrafenib^52^. This study has demonstrated synergistic effects between inhibition of TFF3 (AMPC) and CI-1040 or trametinib, in accordance with the observation of TFF3-mediated activation of the MAPK/ERK pathway. In combination with AMPC, a significantly reduced dosage of the MEK1/2 inhibitors could be administered, potentially reducing toxicity.

Importantly, AMPC exhibited promising efficacy in inhibiting the growth of both the H1975 cell line (with EGFR exon 20 T790M mutation) and the H1299 cell line (with p53 depletion), suggestive of TFF3 inhibition as a potential therapeutic strategy in lung ADC. EGFR tyrosine kinase inhibitors (TKIs) have achieved an excellent clinical response in the subset of patients carrying the sensitizing EGFR mutations, while the acquisition of the secondary EGFR T790M mutation results in acquired TKI resistance^53^. Our study demonstrated that AMPC is efficacious in H1975 cells which are intrinsically resistant to EGFR inhibitors^54^. The synergistic combination of AMPC with MEK1/2 inhibitors in H1975 cells may provide a novel strategy for the treatment of TKI-resistant tumors. In addition, as EGFR is one of the effectors of TFF3^12,55^, a combination approach of AMPC with EGFR TKIs could be explored in lung ADC.

In summary, we have therefore provided preclinical evidence that inhibition of TFF3 in lung ADC may be useful as a treatment for lung ADC either as a single agent or in combination with MEK inhibition.

## Supplementary information

Supplementary information
