# Supplementary material for: A novel small-molecule inhibitor of trefoil factor 3 (TFF3) potentiates MEK1/2 inhibition in lung adenocarcinoma
Source: Oncogenesis. 2019 Nov 4;8(11):65. doi: 10.1038/s41389-019-0173-8 (PMC6828705; doi:10.1038/s41389-019-0173-8)
Supplement: Supplementary file 1 — Supplementary information [file 41389_2019_173_MOESM1_ESM.pdf]

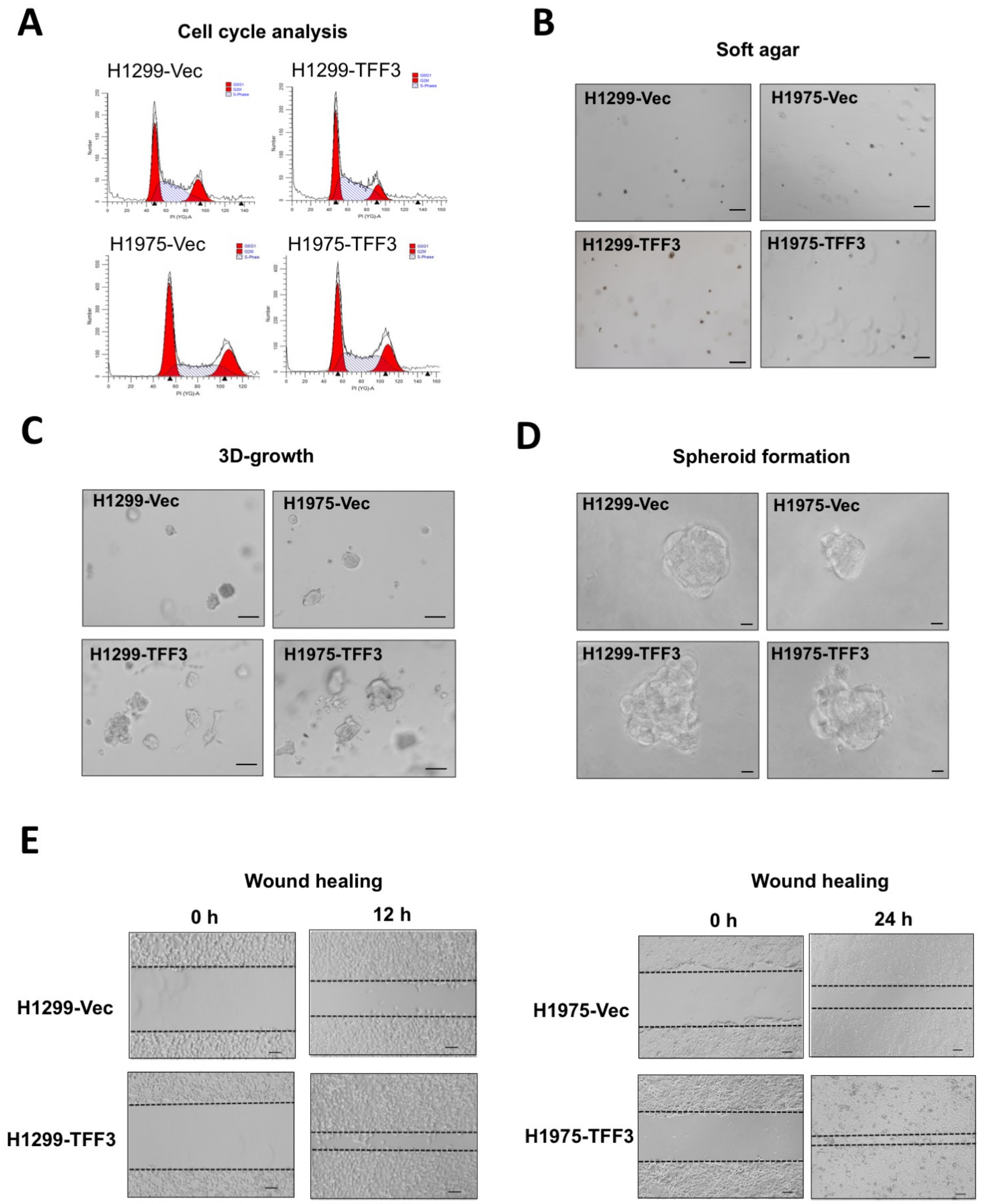

Supplementary Figure 1. Functional effects of forced expression of TFF3 in H1299 and H1975 cells.

A. Cell cycle analysis of H1299/H1975-vec and H1299/H1975-TFF3 cells. The cell cycle histograms corresponding to Fig. 1C were generated using the Modfit software. **B.** Soft agar colony formation of H1299/H1975-vec and H1299/H1975-TFF3 cells. Representative microscopic images of colonies formed by the respective cells in soft agar, corresponding to Fig. 1F, are shown. Scale bar: 200 μm. **C.**

3D Matrigel growth of H1299/H1975-vec and H1299/H1975-TFF3 cells. Representative microscopic images of colonies formed by the respective cells in 3D matrigel, corresponding to Fig. 1H, are shown. Scale bar: 100 µm. **D.** Spheroid formation assay of H1299/H1975-vec and H1299/H1975-TFF3 cells. Representative microscopic images of spheroids formed by the respective cells, corresponding to Fig. 1I, are shown. Scale bar: 20 µm. **E.** Wound healing assay of H1299/H1975-vec and H1299/H1975-TFF3 cells. Images were taken at 12 hours for H1299 cells and 24 hours for H1975 cells. Scale bar: 200 µm.

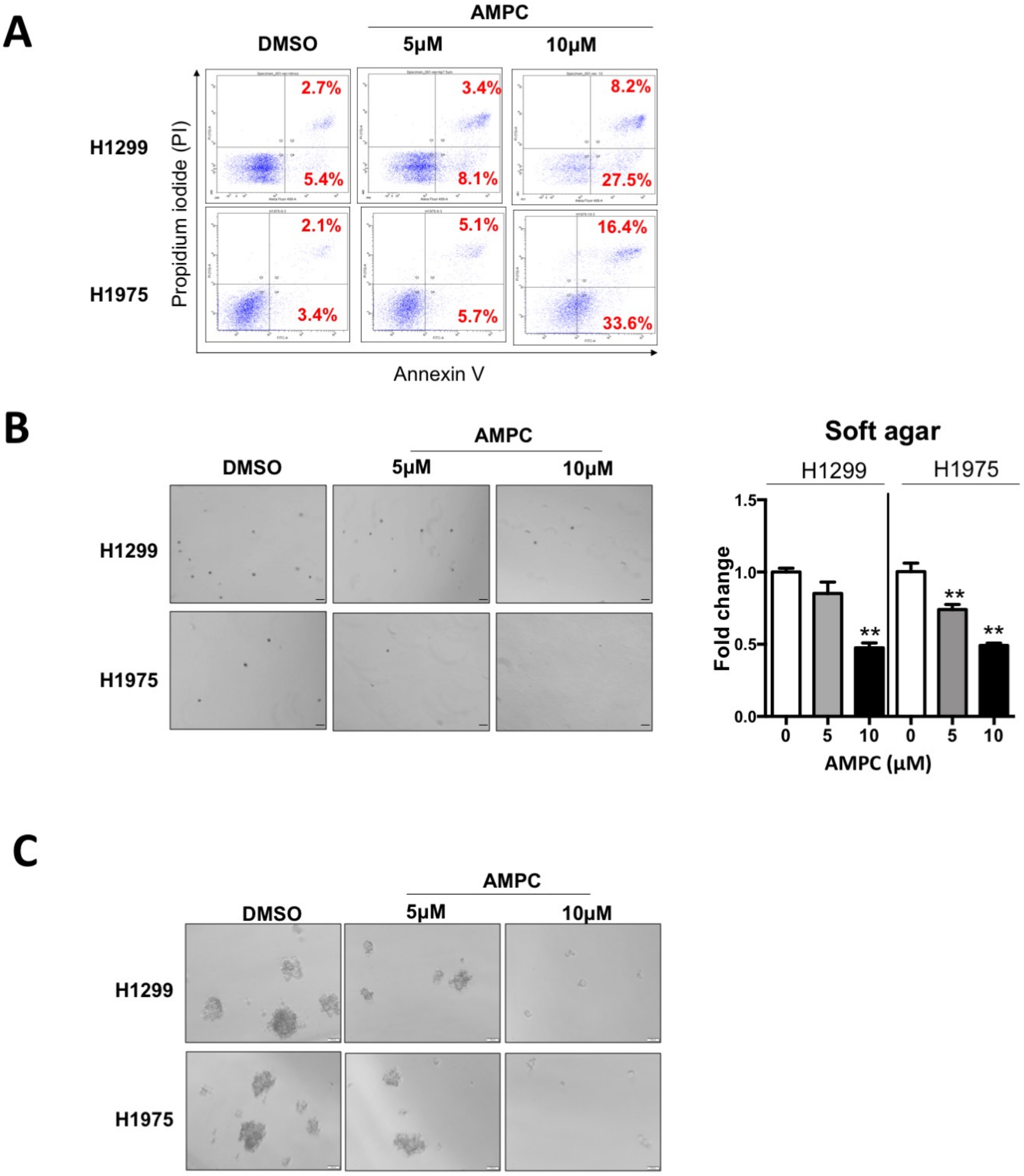

Supplementary Figure 2. Functional effects of inhibition of TFF3 by AMPC in H1299 and H1975 cells.

A. Annexin-V/PI apoptotic cell death assay of H1299 and H1975 cells after 24h AMPC treatment. Flow cytometry plots corresponding to Fig. 3E. **B.** Colony formation in soft agar. H1299 and H1975 cells were seeded in 0.35% agarose in full medium on a base layer of 0.5% agarose and treated with AMPC at the concentrations indicated for 10 days with drug containing media being replaced every 3 days. Colonies formed were counted and fold change in colony numbers relative to the respective vehicle-treated cells are shown in the histogram. Representative microscopic images of colonies formed by the respective cells in soft agar are shown. Scale bar: 200 μm. **C.** Spheroid formation assay of H1299 and H1975 cells after 14 days of AMPC treatment. Representative microscopic images of spheroids formed by the respective cells, corresponding to Fig. 3H, are shown. Scale bar: 100 μm.

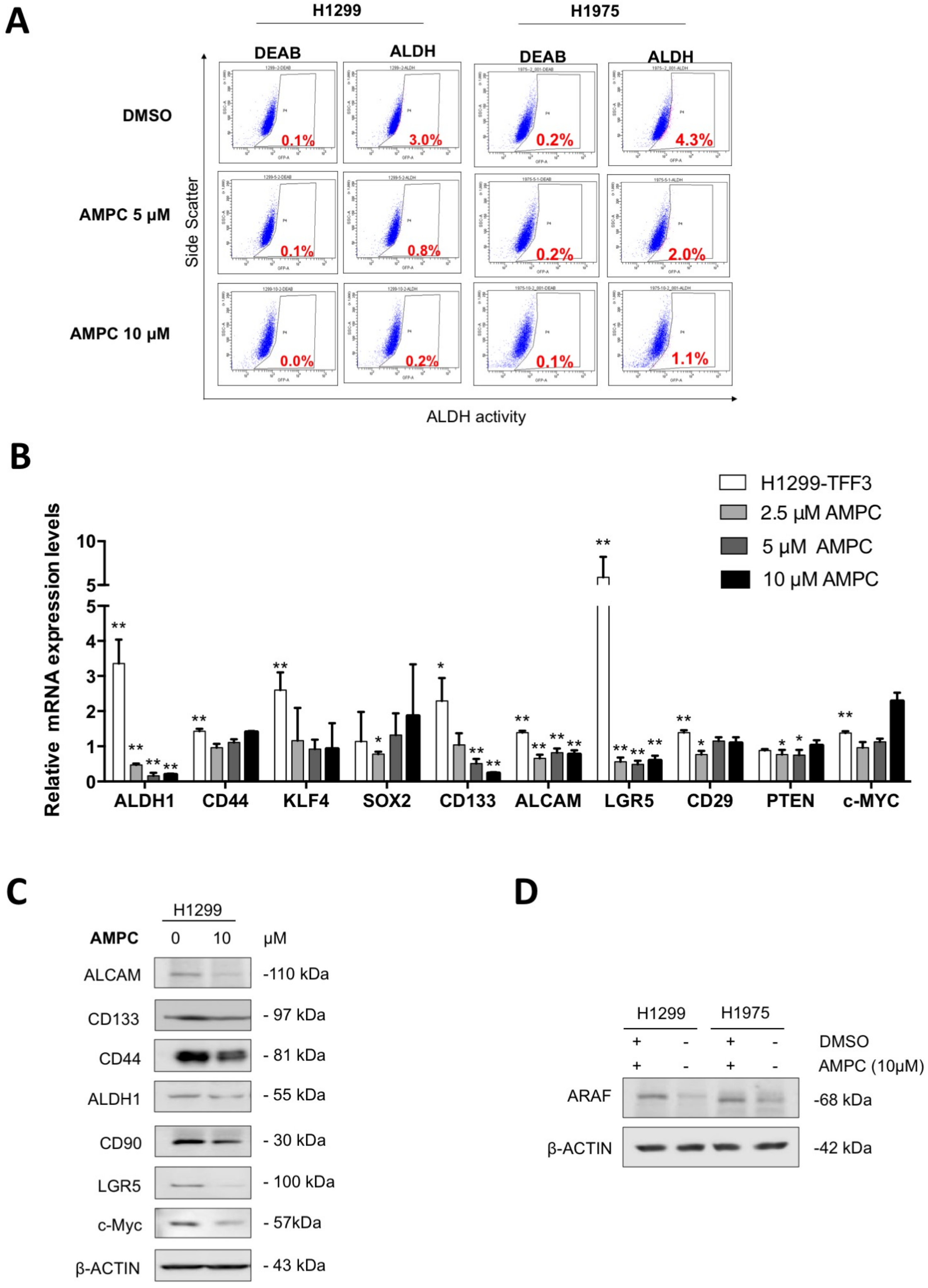

Supplementary Figure 3. Inhibition of TFF3 by AMPC regulates cancer stem cell-related genes in lung ADC cells.

A. ALDEFLUOR^{TM} assay of H1299 and H1975 cells after 24h AMPC treatment at the indicated doses. Representative flow cytometry plots with percentages of ALDH-positive cells, corresponding to Figure 3I are shown. **B.** Expression levels of cancer stem cell-related genes in H1299 cells with forced expression of TFF3 or in H1299 cells treated with AMPC. Relative mRNA expression was detected by qPCR. The ratios are shown in the figure, which indicates increased or decreased gene expression in H1299-TFF3 cells relative to H1299-Vec cells, or AMPC (10μM for 24h) treated H1299 cells relative to DMSO treated H1299 cells. Data are expressed as mean ± SD. *, $p < 0.05$; **, $p < 0.01$. **C.** Western blot analysis of the expression of cancer stem cell-related proteins in DMSO or in H1299 cells treated with 10 μM AMPC for 24 h. β-ACTIN was used as input control. **D.** Western blot analysis of ARAF protein levels in cell lysates of H1299 and H1975 treated with DMSO or 10μM AMPC for 24h. β-ACTIN was used as input control.

Gene Function	Gene	Ratio (TFF3/Vec)	*p value*
**Cell Cycle Control & DNA Damage Repair**	*CDK2*	1.10	3.00E-01
	CDK4	1.39	6.71E-02
	CDK1	0.77	4.29E-01
	CDKN2A	1.83	4.58E-02
	CDKN1A	1.34	1.45E-02
	E2F1	1.18	1.74E-01
	CCND1	1.64	6.98E-05
	MDM2	0.95	2.90E-01
	BRCA1	1.21	3.91E-01
	CYCLIN	1.20	6.35E-01
	CDC25A	1.86	1.66E-01
	CDKN1B	0.87	4.27E-01
	RB1	0.83	6.48E-01
	S100A4	0.80	5.64E-01
	CHEK2	0.94	8.45E-01
**Apoptosis and Cell Senescence**	*HTATIP2*	1.35	3.72E-03
	ARAF	**1272.57**	**8.45E-05**
	BAD	1.00	9.98E-01
	ATM	1.40	5.29E-03
	BCL2	0.81	4.04E-01
	BAK1	0.87	1.50E-01
	BCL2L1	1.38	1.48E-02
	CASP7	1.04	1.35E-01
	RAF	1.03	8.05E-01
	APAF1	1.00	9.46E-01
	BCLAF1	0.96	5.27E-01
	BAK	0.66	5.86E-02
	CFLAR	0.97	9.35E-01
	TERT	0.75	6.04E-01
	TNFRSF1A	0.97	9.65E-01
	TNFRSF25	2.54	9.55E-02
**Adhesion and invasion**	*SNCG*	0.85	3.43E-01
	SERPINE1	2.40	3.66E-01
	PLAUR	2.32	5.05E-05
	VIMENTIN	1.13	6.53E-02
	VEGF	0.94	6.66E-01
	MET	1.74	2.28E-04
	CTNNB	0.79	3.27E-01
	FN1	1.35	4.17E-03
	PLAU	1.13	2.48E-02
	NME1	0.66	1.91E-02
	SERPINE1	2.13	4.27E-01
	MMP1	2.46	2.47E-01
	MMP9	0.81	6.84E-01
	MTA1	2.04	7.67E-02
	TIMP1	1.93	5.07E-01
**Signal Transduction Molecules and Transcription Factors**	*NFKBIA*	0.89	2.55E-02
	STAT3	1.06	7.21E-01
	PIK3R1	1.13	7.40E-01
	JUN	0.83	6.69E-01
	ETS2	1.62	1.26E-01
	STAT5A	0.40	8.00E-02
	STAT5B	1.32	4.80E-02
	AKT	1.60	6.35E-02
	FOS	1.07	5.83E-01
	ERBB2	1.40	5.80E-02
	NFkB1	1.17	6.86E-01
	MYC	0.87	1.47E-01

Supplementary Table 1. qPCR analysis of gene expression changes by forced expression of TFF3 in H1299 cells.

The genes involved in cell cycle control, apoptosis, adhesion and invasion, and signal transduction were examined by qPCR in H1299-Vec and H1299-TFF3 cells. The ratios indicate increased or decreased gene expression in H1299-TFF3 cells relative to H1299-Vec cells. The results are shown as mean and P value of triplicates in a representative experiment.

48h

	H1299			H1975		
CI value	ED_{50}	ED_{75}	ED_{90}	ED_{50}	ED_{75}	ED_{90}
Selumetinib	1.02	1.19	1.50	1.07	0.67	0.42
Pimasertib	0.78	0.94	1.14	0.76	0.85	0.95
CI-1040	0.35	0.43	0.78	0.91	0.75	0.74
Trametinib	1.36	0.99	0.73	0.54	0.42	0.36

72h

	H1299			H1975		
CI value	ED_{50}	ED_{75}	ED_{90}	ED_{50}	ED_{75}	ED_{90}
Selumetinib	0.82	0.87	1.10	1.40	1.05	0.99
Pimasertib	0.89	0.99	1.24	0.68	0.95	1.44
CI-1040	0.64	0.57	0.57	1.09	0.88	0.76
Trametinib	0.56	0.53	0.79	1.18	0.82	0.74

Supplementary Table 2. Combination effects of AMPC and MEK1/2 inhibitors in lung ADC cells.
Combination index (CI) values of AMPC and MEK1/2 inhibitors (Selumetinib, Pimasertib, CI-1040, and Trametinib) are shown in the table. H1299 and H1975 cells were seeded in 96-well plate in media supplemented with 2% FBS and treated with AMPC or MEK1/2 inhibitors alone or combination at the concentration of 0.25-fold, 0.5-fold, 1-fold, 2-fold, and 4-fold of the respective drug's IC_{50}. After 48h and 72h, cell viability was measured by AlamarBlue. CI values were calculated by CompuSyn software based on Chou–Talalay analysis.

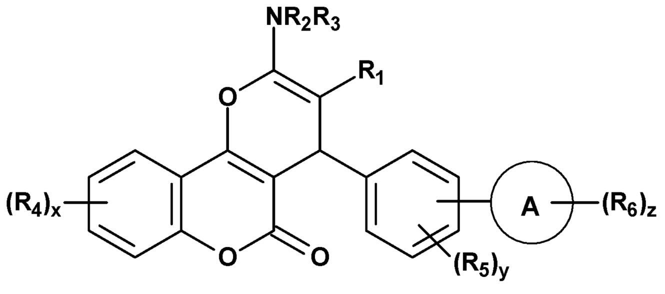

(I)

Supplementary Figure 4. Chemical structure of AMPC.

Compounds useful in inhibiting human trefoil factor 3. More detailed information can be obtained from PCT application SG2018/050277.

https://patentscope2.wipo.int/search/zh/detail.jsf?docId=WO2018226155&recNum=1&office=&queryString=FP%3A%28Peter+Lobie%29&prevFilter=&sortOption=%E5%85%AC%E5%B8%83%E6%97%A5%E9%99%8D%E5%BA%8F&maxRec=47

SUPPLEMENTARY MATERIALS AND METHODS

Antibodies

Protein expression was determined by western blot using following primary antibodies: TFF3 (Abcam ab-108599), β-Actin (Santa Cruz sc-47778), A-Raf (Santa Cruz sc-408), p-MEK 1/2 Ser218/Ser222 (Santa Cruz sc-7995), MEK 1/2 (Santa Cruz sc-436), phospho-p44/42 MAPK T202/Y204 (Cell signaling 4370S), p44/42 MAPK (Cell signaling 4659S), CD44 (Abcam ab157107), ALCAM (Omnimabs OM200093), CD133 (Omnimabs OM118716), ALDH1 (Cell Signaling 54135), CD90 (Omnimabs OM246235), LGR5 (Omnimabs OM283436), and c-Myc (Omnimabs OM209310); Anti-mouse IgG, HRP-linked Antibody (Cell Signaling 7076) and Anti-rabbit IgG, HRP-linked Antibody (Cell Signaling 7074).

Total cell number counting

Cells were plated into 6-well cell culture plate at 2×10^4 cells/well with 10% FBS condition media or 1×10^5 cells/well with 2% FBS condition media. Plates were settled in 37°C in incubator with 5% CO_2 and culture media was changed every two days. Cells were counted every 1-2 days during culture until the sixth day or eighth day. On indicated days, cells in each well were trypsinised with 0.5% trypsin and harvested by centrifuging at 200 × g for 5 minutes. Collected cells were resuspended and counted by haemocytometer.

Cell viability assay

Cells were plated into 96-well cell culture plate at 2×10^3 cells/well and cultured at 37°C in incubator. During the drug treatments, drugs were dissolved in DMSO and added in the 2% FBS condition media while the control groups were added same amount of DMSO. AlamarBlue™ Cell Viability Reagent (Invitrogen) was using to measure cell viability. After the drug treatments for 48h or 72h, culture media was replaced with media containing 10% AlamarBlue reagent. Plates were incubated 4-6 hours and proceeded for plate reading using a fluorescence/ luminesence microplate reader (Tecan). Increased fluorescence was detected by fluorescence using an excitation between 530–560 and an emission at 590 nm.

Cell cycle analysis

Cultured cells after treatment were trypsinized and suspended in 10% FBS medium. After centrifuged for 5 mins, cells pellet was resuspended in 0.5 ml phosphate-bufferedsaline (PBS). Cells were fix by adding 2 ml cold 80% EtOH into cell suspension with vortexing the suspension to prevent clustering of cells during the fixation. Incubation could be 10-15 mins on ice or more at –20°C. Fixed cells were centrifuged and washed with 2 ml PBS. Finally, cell pellet was suspended in 500 µL staining solution (50 µg/ml RNaseA and 0.1% Triton X-100 in PBS) and incubated for 30-40 min at room temperature. Propidium Iodide (PI) (10 µg/mL, from the stock) was added directly to the cell solution and vortex gently. Samples were incubated at least 5 min at room temperature before fluorescence-activated cell sorting (FACS) analysis. Results were analyzed by Modfit software and population of G1, S, and G2 phase was compared between samples.

Cell apoptosis measurement

Apoptotic cell death was determined using Annexin-V AlexaFluor® 488 Propidium Iodide (PI) Dead Cell Apoptosis Kit (Life Technologies) following the recommended protocols. Cells were seeded in 6-well plates at the density of 2×10^5 cells/well and incubated 8 hours for cells to attach to the plates. Apoptosis was induced by serum-free starvation or drug treatment for 24 hours or 48 hours. Cells were harvested, washed with PBS, and suspended in 100 µL 1X Annexin-binding buffer. After that, 100 µg/mL working solution of PI and 5 µL of FITC Annexin V was added to each sample and incubated for 15 mins. For single stain control samples, only PI or Annexin V was added. Another 400 µL 1X Annexin-binding buffer was added into samples after the incubation. Samples were kept on ice and analyzed by flowcytometry as soon as possible. The population was separatde into three groups: live cells with low level of fluorescence, early apoptotic cells with green fluorescence, and late apoptotic cells with both red and green fluorescence.

Apoptotic cell death was also determined using Caspase-Glo caspase 3/7 kit (Promega Madison, WI) following the recommended protocols. Cells were plated at a density of 1×10^4 cells/ well in white-walled 96-well plates. After serum-free starvation or drug treatment for inducing apoptosis, prepared Caspase-Glo 3/7 reagent was added into per well at the ratio of 1:1 with the culture media. Reagent and culture media were mixed at 300–500 rpm using a plate shaker for 30 seconds and followed by a 1-hour incubation at room temperature in dark. Finally, luminescence of each well was measured with the fluorescence/ luminesence microplate reader (Tecan, Zurich, Switzerland).

Anchorage-independent growth

Assays including colony formation and foci formation were performed to investigate anchorage-independent growth of ADC cells. For colony formation, each well of a 24 well plate was covered with a base agar layer of 0.5% agarose in serum free medium. Cells re-suspended in 0.35% agarose and culture medium was added to each well on the top of the base agarose. Normal culture media was added on the top of each well and refreshed every three days. After two weeks of culture, pictures of cell colonies were taken under microscope and colony numbers for each well were counted at 40 × magnification (Olympus, Tokyo, Japan).

For foci formation, 1×10^3 cells were seeded in 6-well plates and cultured for two weeks. Cold methanol was used for fixing the cell. After leaving the plates open to dry for two hours, 1% crystal violet in 20% EtOH was added into the well. Cell foci were stained in purple color with crystal violet. Pictures were taken for each well.

Three-dimensional culture assay

Cells were grown in three-dimensional culture on a layer of growth factor-reduced Matrigel (BD Biosciences) in 96 well plate. First, 40μL Matrigel was added to coat the surface of cell culture plate. After that, 1×10^3 cells were cultured in 4% Matrigel/medium solution upon the Matrigel base for 9 days. After 9 days, pictures of cells colonies were taken under microscope and stained with AlamarBlue to examine the cell viability. 10 μL of AlamarBlue was added to each well, and left for 4 hours in the CO_2 incubator before spectrofluorometric reading were taken at 560nm/590nm. For the drug treatment experiments, cells were growth in 4% Matrigel for three days without drugs. At the fourth day, half of the 4% Matrigel/medium solution in the plates was removed with the same amount of solution with drugs or DMSO. Later, the medium solution was refreshed every two days until the tenth day.

Migration and invasion assay

In vitro cell migration and invasion assays were performed using 24-well cell invasion chamber (8 μm pore size) following the provided protocol (BD Biosciences). For invasion assessment, inserts were coated with 10% Matrigel before seeding the cells. Cells (2×10^4 cells) were seeded with serum free medium in the upper chamber and complete medium were added in the bottom well. After 12h incubation for migration and 24h incubation for invasion, cells on the lower surface of the inserts were washed with PBS and fixed with 4% paraformaldehyde. After removing the non-migrating or non-invading cells on the upper surface of the inserts with cotton tipped swab, the cells on lower surface of

inserts were stained with Hoechst 33342 (Sigma). The migrated and invaded cells were observed and counted using fluorescence microscope.

For the wound healing assay, cells were cultured in a 6-well plate until 100% confluence. Three perpendicular equal straight artificial wounds were drawn using a pipette tip. Cells were maintained with 10%FBS culture medium and pictures were taken at 0, 12, and 24h after drawing the wounds.

Histological analysis

Morphologic analyses were carried out on 4% paraformaldehyde-fixed for 24 to 48 hours before being processed and embedded into paraffin wax. Deparaffinized, rehydrated sections were immunolabeled with anti-Ki67 antibody (Abcam ab-15580) and anti-Trefoil factor3 (TFF3) antibody (Abcam ab-108599) and stained with hematoxylin (Goodbio technology, Wuhan, China). The antigen antibody was visualized using 3,3-diaminobenzidine. Sections were then dehydrated, cleared with xylene, and mounted with resinous mounting medium. In every tumor section, the percentage of Ki67 positive cells in three different regions was counted. The percentage of positive TFF3 staining area was conducted with Image J software, and calculated by the following formula:

The percentage of TFF3 = TFF3 staining positive area÷Region area×100%

TUNEL Staining Assay

Cell apoptosis was detected through Terminal Deoxynucleotidyl Transferase dUTP Nick-end Labeling (TUNEL) assay using a TUNEL Apoptosis Detection Kit (Alexa Fluor 488) (YeSen Biotechnology, Shanghai, China) as per manufacturer's instruction. The relative fluorescence intensity was calculated as the ratio of the mean fluorescence intensity of the TUNEL staining to that of DAPI staining. Two fields per section of four independent sections in each group were evaluated.
